# Dentistry One Hundred Years Ago

**Published:** 1892-05

**Authors:** William Dunn

**Affiliations:** Florence, Italy


### DENTISTRY ONE HUNDRED YEARS AGO.

BY WILLIAM DUNN, JR., D.D.S., FLORENCE, ITALY.

A QUAINT and instructive old book is that written by a certain Antonio Campani, "public dentist of the city of Florence." Its title is as follows:

Odontologia. | ossia | Trattato sopra i denti | Opera | di Antonio Campani. | Pubblico Dentista Fiorentino | Nella quale si ragiona dei mali | dé denti, e loro cura, e specialmente della maniera di estrarli. | Firenze MDCCLXXXVI | per Gaetano Cambiagi | Stampator Granducale.—con licenza dei Superiori.

It is now a very rare work; the only copy I know of, dusty and bound in parchment, but still in excellent preservation, being the property of Signor Cianchi, surgeon-dentist in this town, and a distant relation of the author.

The book was edited by one Cambiagi, in 1786, so that it is over one hundred years old.

Of the author himself we are acquainted in a rather diffuse autobiography prefaced to the work: Born in Florence, he seems from the first to have been surrounded by professional influences, for his father was also a dentist, and kept him at work as soon as he left school.

At the age of twenty he must have become weary, for he enlisted, and soon after his regiment, much to his chagrin, was ordered off to Poland and Moravia to fight the Germans. When he returned, wounded, to his native city, the public dentist who was his predecessor, one Larini, died very opportunely, and Campani begged the Grand Duke Francis I. to grant him the place of the defunct professor "for the benefit of the public."

In the first part of the book, which he considers the more important, the author treats of the qualities necessary in a dentist to be successful, and the first condition he lays down is that he must be a young man of spirit, full of courage, with a compassionate heart but a cruel hand. He must be able to inspire confidence and know everything by heart which concerns his profession, for, if asked questions by "people of quality," he must be able to answer like an intelligent professor, and not like an ignorant charlatan.

Ideas on embryology are not quite up to date. According to him, the enamel is deposited from the inner surface of the alveolar sack *onto* the tooth-germ, in the shape of a "humour" which consolidates.

Perhaps the most interesting chapter in the book is the one which treats of the causes of decay: Starting from the theory that the teeth are bones, he remarks they are not covered by flesh, like other bones; and the enamel is not sufficient protection for the fluids constantly circulating in the roots of the teeth, fluids which, under the influence of heat and cold, condense and coagulate, and thus decompose and soften the tooth substance.

Teeth decay more quickly if the fluids they contain are very dense or badly constituted, as they stagnate more easily.

Among other causes of decay are a bad digestion, any passion that will dry up or modify the blood, the exposure of the head to night-air, and, lastly, the remains of food between the teeth.

Tartar is not only due to acid eructations and emanations from the stomach, but also to the descent of vitiated humors from the brain (*sic!*).

A large part of the work is devoted to extraction, which is performed usually with the old key, but also with levers and pincers invented by the author, and which he describes with copper-plate illustrations.

It is curious to see how much trouble he takes to describe which teeth may be dexterously and easily removed, and how an ugly-looking tooth or root is best left alone, so as not to endanger the life of the patient and the honor of the professor.

Of prophylaxis and dental hygiene not a word is said; but two systems of the treatment of decay are described,—filing, for which he indicates small files of various shapes, all of which he illustrates, and stopping.

It is worthy of note that, in speaking of files and keys, he says the instruments must be got from England, where they were made in greater perfection than elsewhere.

Stopping was done with lead-foil. Teeth decayed on the masticating or buccal surface were good for stopping, but proximal cavities were hopeless, for the "air would still pass through, even after the plugging was completed." The cavity was cleansed from food and saliva with a probe, and then a lead-foil pellet was pressed in, first with the finger, then with a ball-burnisher, which also served to smoothen the surface.

When a patient "of quality" came along, it was customary to lay a gold-foil pellet on the floor of the cavity; but the completion with lead-foil was deemed necessary.

When inserted by an expert hand, such a filling sometimes lasted several years.

An exposed pulp was cauterized, and the instructions given were to place the operating-chair near a wall, so that the patient might be steady, and not tip the chair backward on the operator. Then a suitable instrument was taken and made red-hot in the flame, and immersed in the pulp three or four times.

A certain *naïveté* comes out when the author says that if the tooth still gives trouble, after all the different processes have been tried, it showed that the disease had reached a point that would not admit of any cure, and then extraction would be the only course open. Doubtless, when the patient had gone through operations like the actual cautery, he must have come to the same conclusion.

A few more chapters are devoted to prosthetic dentistry. The artificial teeth or sets were made of ivory, and tied to the adjoining natural organs with catgut or silk. Another way he had of attaching sets when the alveolar processes were well pronounced was by springs, which clasped the processes on the palatal and labial side.

Some of the certificates left to Campani by grateful patients are so exquisite that we will close with the translation of one of them. It is from a Venetian prelate, who had been suffering for some time with alveolo-dental abscess with external fistulous opening; and after consulting no end of "professors" in this, as well as in other cities, came to Campani on the 28th of September, 1780:

". . . But notwithstanding all the persuasions of my friends," writes he, "I confess that I had not courage enough to have the tooth pulled; till one day, accompanied by two of my friends, who witnessed the operation, and who undersign their names, I decided to come to Professor Campani, who extracted my tooth, by many others declared to be a dangerous one, with such delicacy that I scarcely experienced any pain; and at present, thanks first to God, and secondly to the most skilful Professor Campani, to whom I protest myself eternally grateful, I am in perfect health again."

The signatures of patient and witnesses follow, accompanied by the sacramental m. p. (manû propriâ); and doubtless their grandsons walk the streets of Florence to this very day.

